# Comparing the post-operative outcomes of two extra-oral surgical approaches for sialoadenectomy: a randomised case-control study

**DOI:** 10.1017/S0022215125103617

**Published:** 2025-12

**Authors:** James Onuorah Akpeh, Uchenna C Okechi, Karpal Singh Sohal

**Affiliations:** 1 Department of Otolaryngology, College of Medicine, University of Nigeria, Enugu, Nigeria; 2 Department of Oral and Maxillofacial Surgery, College of Medicine, University of Nigeria, Enugu, Nigeria; 3 Department of Oral Health Services, Muhimbili National Hospital, Dar es Salaam, Tanzania

**Keywords:** facial nerve, outcomes, randomised control trials, salivary gland, surgical techniques/endoscopy

## Abstract

**Objectives:**

This study aimed to compare the outcome of the standard trans-cervical approach and modified trans-cervical approach regarding cosmesis and complications outcomes in a tertiary hospital in Nigeria.

**Methods:**

In this study, 25 patients with submandibular salivary gland lesions adjudged not to be malignant neoplasia were included. They were randomised into the two groups by balloting method.

**Results:**

Twelve (48 per cent) patients had the traditional transcervical approach while 13 (52per cent) had the modified approach. There was no statistically significant difference between the groups in terms of general complication, transient paresthesia and wound infection (*p* > 0.05). The presence of a non-visible scar was reported in almost 85 per cent of patients in the modified trans-cervical approach group compared to 50 per cent in the standard trans-cervical approach group.

**Conclusions:**

Though by observation the modified trans-cervical approach was superior to the standard trans-cervical approach, the differences were statistically insignificant.

## Introduction

Submandibular salivary glands (SMG) are major salivary glands within the head and neck region. They are located in the submandibular triangle, underlying the investing layer of deep cervical fascia.^1^ This gland contributes to the production of saliva which is important for the initiation of food digestion together with lubrication of the mouth during feeding and speech.^2^ Saliva also serves as a buffer to prevent dental caries by promoting remineralisation.^3^

Several disease conditions like neoplasms and infections can affect the submandibular salivary gland which may warrant surgical excision of the gland.^4^ Surgical access approaches to the submandibular gland have evolved over the years due to the need to achieve cosmesis and avoid neural complications.^4^ Traditionally, access to this gland was extensive to achieve adequate exposure; however, due to the need for better cosmetic outcomes, more minimal techniques, like endoscopic and robotic techniques, are currently being researched.^4^

Approaches to the SMG could be intraoral (trans-oral) or extra-oral. Extra-oral approaches include post-auricular, lateral trans-cervical (TCA) and submental. Each of these approaches has its complications and risks. The access incision can be important as it could be associated with better cosmesis, reduced scar tissue or keloid formation, minimal blood loss, shorter hospital stay and ultimately affect patients’ finances.^4^

Minimum access surgeries with endoscopy though beneficial, are underutilised by most surgeons in African settings including head and neck surgeons. Some reasons for this include inadequate facilities, the absence of specialist endoscopy surgeons the lack of dedicated endoscopy units and theatre time.^5^ Taking this into account and the fact that the TCA is most preferred for the excision of the submandibular gland, in our facility, a modified TCA (MTCA) has been used. The benefits of the MTCA over TCA are controversial, as it is unclear whether any difference in the post-operative outcome between these two approaches exists. Thus, this study aimed to compare the outcome of these two extra-oral surgical approaches in terms of cosmesis and complications outcomes in a tertiary hospital in Nigeria. To the best of the authors’ knowledge, no study has been done on this topic in Nigeria. It is hoped that the study will add to the corpus of knowledge on this subject matter.

## Methods

### Patient selection

**Figure 1.**
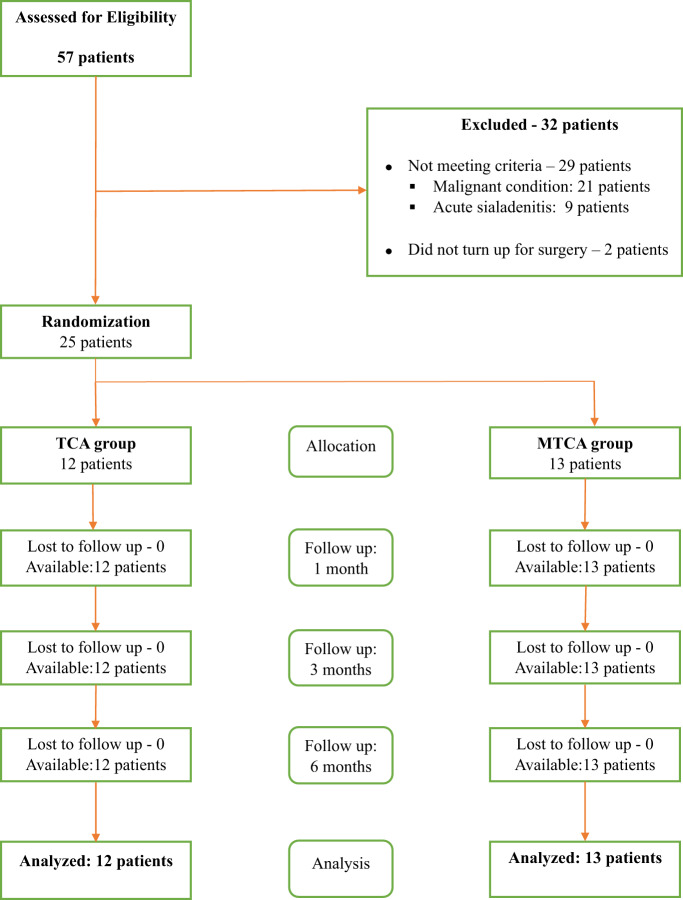
CONSORT diagram.

In this prospective randomised control study carried out for three years (January 2021 to December 2023), patients were recruited from the Otorhinolaryngology and Oral and Maxillofacial Surgery clinics of the University of Nigeria Training Hospital (UNTH). Patients diagnosed with non-malignant submandibular salivary gland lesions and approved for surgical treatment options were the main eligibility criteria for inclusion in this study. In this study, only patients with non-malignant submandibular gland lesions whose only treatment would be sialoadenectomy were included (Figure 1). Patients with a history of keloid or hypertrophic scar formation well as those with uncontrolled systemic disease like diabetes mellitus were excluded from the study.

### Randomisation

A balloting technique where each patient recruited for the study was asked to select an envelope from an opaque bag containing two envelopes. This was administered by a resident doctor who was not detailed on the study protocol and was used to randomly assign each patient to one of the two surgical treatment groups with equal probability: (1) standard trans-cervical approach (TCA) - operated with the standard TCA, (2) modified TCA (MTCA) - operated with a modified TCA. The patients were allocated to the corresponding surgical access approach they picked.

### Pre-surgical preparation

All patients with the diagnosis of a benign submandibular tumour (e.g., pleomorphic adenoma) and inflammatory disease of the submandibular gland (chronic sialadenitis, intraglandular sialolithiasis) were assessed for surgical fitness by a team of anesthesiologists. The pre-operative investigation included; complete blood counts, biochemical tests (serum electrolytes, liver and renal function tests) and imaging of the affected submandibular gland using a contrast enhanced CT scan. Cases without clear evidence of pathology in the gland like enlargement, discharge, pain and presence of opacities where excluded.

### Surgical technique

**Figure 2.**
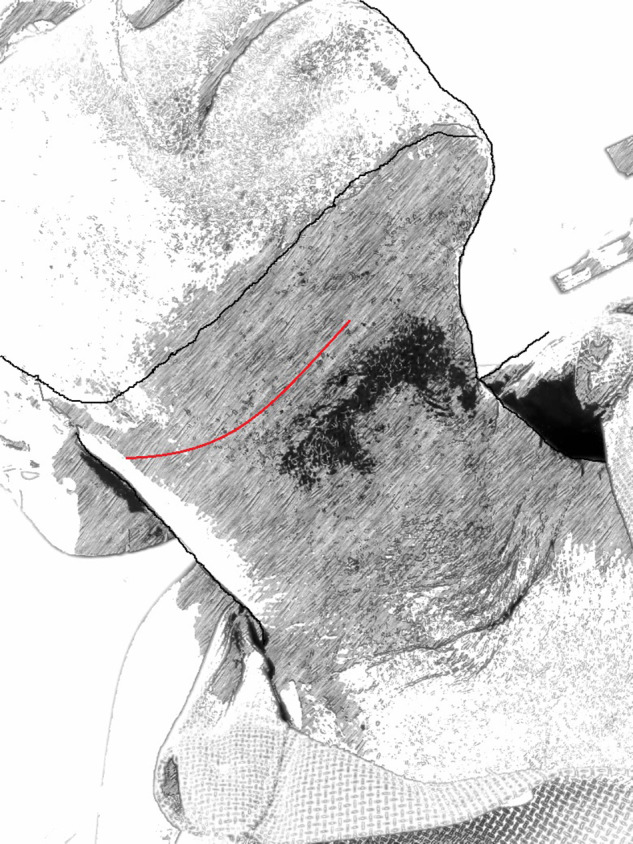
A schematic presentation of the standard trans-cervical approach.

All surgical procedures were carried out under general anesthesia. The surgeries were performed by one of the two lead surgeons (one otorhinolaryngologist and one oral and maxillofacial surgeon) after going through and agreeing on the protocol and both surgeons performed identical surgeries. The description of surgical approaches is as below:

The standard TCA to the submandibular salivary glands which has withstood the test of time used in this study was made following the natural skin crease of the neck at least 2-3 cm below the lower border of the mandible. The length of the skin incision is usually unlimited but could generally be about 6-7 cm in length (Fig. 2).

**Figure 3.**
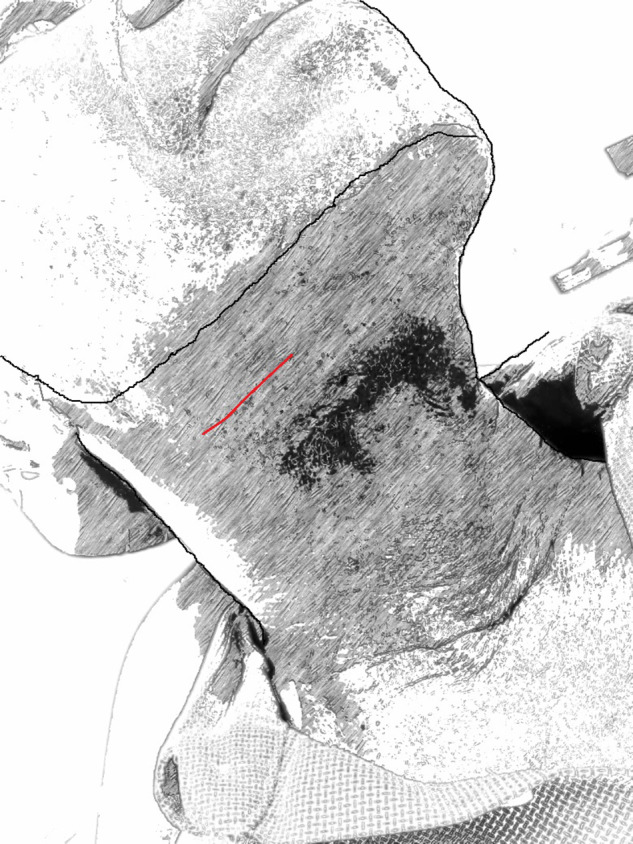
A schematic presentation of the modified trans-cervical approach.

The MTCA used in this study is similar to the former but is shorter in length. It extends from an imaginary line corresponding from the first premolar to the first molar and measures about 4 cm long (Fig. 3).

### Follow-up and clinical outcome

The outcome measurements being compared between the two surgical approaches/techniques groups were the post-operative complications and the aesthetic of the surgical wound. Follow-up assessment of the patients was done at one month, three months and six months post-operatively. Scar visibility was assessed using a visual analog scale of 0 to 9 self-administered by the patient.

### Collected data

Information related to the diagnosis, age, sex of the patients, the side affected, presenting complaint, duration of presentation, surgical approach used, post-operative complications and outcomes recorded for each patient.

### Data analysis

Data coding and analysis were done using the Statistical Package for Social Sciences software (SPSS) for Windows (version 27, Armonk, New York: IBM Corp). The age of the patients was dichotomised into young adults (≤ 45 years) and older adults (> 45 years). Scar visibility was assessed using a visual analog scale of 0 to 9 self-administered by the patient. A score of 0 to 3 is considered NIL, 4 to 6 PARTIAL and 7 to 9 as FULL. The scar visibility was reported at six months post-operative visit. Means, standard error of the mean and proportions were used for descriptive analysis. The chi-squared test and Fisher’s test were used where applicable for the grouped data, and the probability level of α less than 0.05 was selected for statistical significance.

### Consent

The present study was conducted according to the tenets of the Declaration of Helsinki. The research protocol and informed consent/assent forms were approved by the Health Research Ethics Committee of the UNTH. Written informed consent was obtained from each patient.

## Results

This study included 25 patients (15 males and 10 females) with a mean age of 46.8 years (SEM = 2.70) and an age range from 27 years to 79 years. Twelve (48 per cent) patients were operated on through the standard TCA, and 13 (52 per cent) underwent sialoadenectomy using the MTCA. The majority (18, 72 per cent) had a pre-operative diagnosis of inflammatory disease (13 chronic sialadenitis and 5 sialolithiasis), and the remaining 7 (28 per cent) had a benign salivary gland lesion (pleomorphic adenoma).

There was no statistically significant difference between the patients in either study group in terms of age, sex and pre-operative diagnosis (Table 1). All 25 patients were followed up for six months post-operatively without any dropouts.

**Table 1.** Characteristics of the patients enrolled in this study

Variable	Surgical technique		*p*-Value
	TCA (*n* = 12)	MTCA (*n* = 13)	
Sex			
Male	8 (66.7%)	7 (53.8%)	0.688
Female	4 (33.3%)	6 (46.2%)	
Age groups			
≤ 45 years	6 (50.0%)	7 (53.8%)	0.848
> 45 years	6 (50.0%)	6 (46.2%)	
Mean age (years) ± SEM	47.6 ± 3.76	46.0 ± 3.99	0.776
Diagnosis			
Inflammatory	7 (58.3%)	11 (84.6%)	0.202
Benign	5 (41.7%)	2 (15.4%)	

MTCA = modified trans-cervical approach; SEM = standard error of the mean; TCA = standard trans-cervical approach.

During the entire post-operative follow-up period, 14 (56.0 per cent) patients had one or more complications. Generally, eight (32 per cent) patients had transient paresthesia, two (8 per cent) patients had surgical site infection and self-reported scarring at six months follow-up was recorded in eight (32 per cent) patients. Only four (16 per cent) had more than one complication, whereas three patients had transient paresthesia and scarring and one had surgical site infection and scarring. All the patients with scarring had partially visible scars on the visual analog scale (VAS) scale. The mean VAS score for MTCA was 1.92 (SEM=0.43) and for TCA was 3.58 (SEM = 0.434), and the difference in mean score was statistically significant (*p* = 0.012).

There was no statistically significant difference between the groups in terms of general complication, transient paresthesia and wound infection (*p* > 0.05). The presence of a non-visible scar was reported in almost 85 per cent of patients in the MTCA group compared to 50 per cent in the TCA group; however, the difference was statistically insignificant (Table 2).

**Table 2.** General and specific complications during the post-operative period following sialoadenectomy

Variable	Surgical technique		*p*-Value
	TCA (*n* = 12)	MTCA (*n* = 13)	
General complication			
No	4 (33.3%)	7 (53.8%)	0.428
Yes	8 (66.7%)	6 (46.2%)	
Transient paresthesia			
No	8 (66.7%)	9 (69.2%)	0.999
Yes	4 (33.3%)	4 (30.8%)	
Surgical site infection			
No	11 (91.7%)	12 (92.3%)	0.999
Yes	1 (8.3%)	1 (7.7%)	
Scarring at six months			
No	6 (50.0%)	11 (84.6%)	0.09
Yes	6 (50.0%)	2 (15.4%)	

MTCA = modified trans-cervical approach; TCA = standard trans-cervical approach.

## Discussion

We conducted the first randomised clinical trial (RCT) of submandibular gland sialoadenectomy to accurately evaluate the differences in post-operative outcomes between the TCA and MTCA. In this study, only patients with benign salivary gland tumours and chronic salivary gland inflammatory conditions were included. This is because they both require almost similar surgical management, unlike malignant disease, which entails more radical resection of tissues together with neck dissection.

The findings of this study depicted no significant difference in the distribution of patients in either group (TCA vs MTCA) by age, sex and diagnosis. Such findings indicate that these variables had no significant impact on the outcome of the surgery. Chronic sialadenitis and pleomorphic adenomas were the major reasons for submandibular salivary gland excision. This finding is similar to findings in previous reports.^6^^,^^7^ Findings from this study augment other reports that total sialoadenectomy is still a common management practice for benign and inflammatory conditions of the salivary glands,^6^^–^^8^ despite Ge *et al*.^9^ advocating for partial sialoadenectomy of the submandibular gland as treatment of benign salivary gland conditions.

There was no statistically significant difference in the occurrence of general post-operative complications between the two approaches in the current study. Complications that were noted in this study were similar to reports in the literature.^10^^,^^11^ At six months post-operatively, it was noted that the majority of patients who were in the MTCA had a minimum or no scar visibility compared to the TCA group though the difference was statistically insignificant. However, the small sample size of the study might be attributable to this insignificant difference.

The MTCA used in our setting was 4 cm in length, unlike Hussain and Murray.^10^ who used 2.5–3 cm length incision but in a similar location to ours. The reasons for opting for the MTCA were avoiding unnecessary sacrifice of the facial vessels and minimising tissue injury using the overgenerous length of the incision.^10^ Our findings depict that it is possible to successfully perform submandibular salivary gland excision using a 4 cm incision with outcomes similar to those of standard TCA. Though we believe our modified technique has improved overall cosmetic outcomes; however, either approach is safe to use depending on the skills/experience of the surgeon.

The current study has some limitations that are worth mentioning. First, this RCT excluded patients with malignant conditions of the submandibular gland. Hence, the results of this research portray benign and inflammatory conditions of the submandibular salivary gland rather than all disease conditions. Second, the sample size used was small due to the rarity of the disease condition in our setting, and a larger sample could have given a better comparison of the groups. Lastly, we did not consider the difference in time of surgery between the two groups, hence we could not measure the cost-effectiveness of the approaches. Thus, a multicentric RCT involving a larger sample size is recommended to ascertain the advantage of either approach over the other.

- Chronic sialadenitis and pleomorphic adenomas were the major reasons for submandibular salivary gland excision
- There was no statistically significant difference in the occurrence of general post-operative complications between the two approaches (standard trans-cervical [TCA] vs modified trans-cervical [MTCA]) in the current study
- MTCA is useful in avoiding the sacrifice of the facial vessels and minimising tissue injury using the overgenerous length of the incision

## Conclusion

This is the first RCT comparing two surgical approaches for submandibular gland sialoadenectomy, and it found that, by numbers, the MTCA was superior to the standard TCA, the differences were statistically insignificant. Therefore, either approach is safe.
